# Effects of regular sauna bathing in conjunction with exercise on cardiovascular function: a multi-arm, randomized controlled trial

**DOI:** 10.1152/ajpregu.00076.2022

**Published:** 2022-07-04

**Authors:** Earric Lee, Iiris Kolunsarka, Joel Kostensalo, Juha P. Ahtiainen, Eero A. Haapala, Peter Willeit, Setor K. Kunutsor, Jari A. Laukkanen

**Affiliations:** ^1^Faculty of Sport and Health Sciences, University of Jyväskylä, Jyväskylä, Finland; ^2^Natural Resources Institute Finland (Luke), Joensuu, Finland; ^3^Institute of Biomedicine, School of Medicine, University of Eastern Finland, Kuopio, Finland; ^4^Clinical Epidemiology Team, Medical University of Innsbruck, Innsbruck, Austria; ^5^Department of Public Health and Primary Care, University of Cambridge, Cambridge, United Kingdom; ^6^National Institute for Health Research Bristol Biomedical Research Centre, University Hospitals Bristol and Weston National Health Service Foundation Trust and the University of Bristol, Bristol, United Kingdom; ^7^Translational Health Sciences, Bristol Medical School, University of Bristol, Southmead Hospital, Bristol, United Kingdom; ^8^Central Finland Central Hospital, Department of Internal Medicine, Jyväskylä, Finland; ^9^Institute of Clinical Medicine, University of Eastern Finland, Kuopio, Finland

**Keywords:** blood pressure, cardiorespiratory fitness, exercise, heat therapy, sauna bathing

## Abstract

Regular exercise and sauna bathing have each been shown to improve cardiovascular function in clinical populations. However, experimental data on the cardiovascular adaptations to regular exercise in conjunction with sauna bathing in the general population are lacking. Therefore, we compared the effects of exercise and sauna bathing to regular exercise using a multi-arm randomized controlled trial. Participants (*n* = 47) aged 49 ± 9 with low physical activity levels and at least one traditional cardiovascular disease (CVD) risk factor were randomly assigned (1:1:1) to guideline-based regular exercise and 15-min postexercise sauna (EXS), guideline-based regular exercise (EXE), or control (CON) for 8 wk. The primary outcomes were blood pressure (BP) and cardiorespiratory fitness (CRF)_._ Secondary outcomes included fat mass, total cholesterol levels, and arterial stiffness. EXE had a greater change in CRF (+6.2 mL/kg/min; 95% CI, +4.2 to +8.3 mL/kg/min) and fat mass but no differences in BP when compared with CON. EXS displayed greater change in CRF (+2.7 mL/kg/min; 95% CI, +0.2 to +5.3 mL/kg/min), lower systolic BP (−8.0 mmHg; 95% CI, −14.6 to −1.4 mmHg), and lower total cholesterol levels compared with EXE. Regular exercise improved CRF and body composition in sedentary adults with CVD risk factors. However, when combined with exercise, sauna bathing demonstrated a substantially supplementary effect on CRF, systolic BP, and total cholesterol levels. Sauna bathing is a valuable lifestyle tool that complements exercise for improving CRF and decreasing systolic BP. Future research should focus on the duration and frequency of exposure to ascertain the dose-response relationship.

## Graphical abstract

**Figure:**
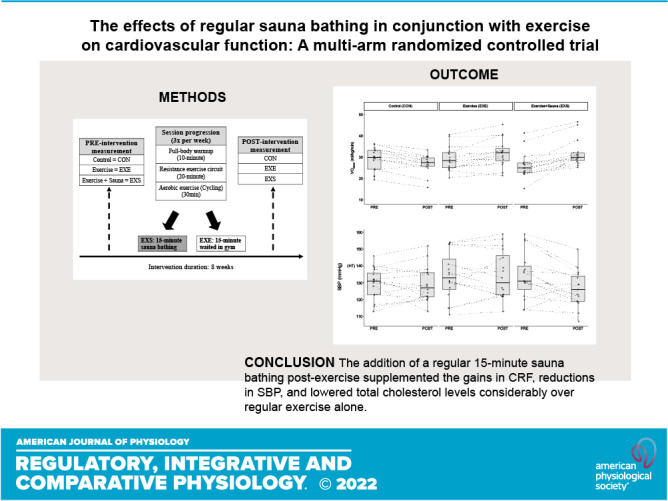

## INTRODUCTION

Physical activity and exercise training are well-documented strategies to prevent ailments (1) and various diseases (2). The current health and exercise guidelines (3) recommend 150–300 min of moderate-intensity physical activity spread across three to five sessions per week. In addition, resistance exercise should be performed at least twice a week (3, 4). Evidently, we have come to a firm understanding of exercise and how it can be used to improve cardiovascular health. However, unlike exercise, heat therapy and the health benefits of Finnish sauna bathing are still not well understood, despite its increasing use throughout the world (5), even though observational cohort studies (6–8) have found regular use of the sauna to be positively associated with numerous cardiovascular outcomes.

Indeed, most studies investigating the efficacy of sauna bathing have either been acute (0–30 min after sauna) or short-term (2–4 wk long) (9), and as pointed out by a recent review, long-term experimental evidence in heat therapy (10) based on the Finnish sauna is needed. The efficacy of sauna use on a regular basis when combined with exercise has been shown in both extremes of the population; in well-trained cyclists (11) and runners (12), as well as patients with heart failure (13) and other diseases (14). However, these data are somewhat limited for the general population.

A significant portion of the general population today has at least one cardiovascular disease (CVD) risk factor (obesity, elevated blood pressures, elevated cholesterol, family history of coronary heart disease, and smoking or a history of smoking). This includes the majority of the population in Australia (15), Canada (16), Europe (17), United States (18), and a substantial percentage of people in China (19). This underscores the problems of public health in our modern society. Thus, it is important to develop interventional strategies that target these groups, as they form a larger part of the general population. Furthermore, these groups often stand to benefit most from lifestyle-related interventions (20).

One traditional CVD risk factor that warrants consideration is elevated blood pressure (BP) level, as increases in BP have been associated with an increase in CVD risk (21). BP has also been well documented to respond favorably to regular physical activity, which plays a pivotal role in the nonpharmacological management of hypertension (22). However, recent evidence suggests that regular heat therapy can lower BP to a comparable, if not larger degree (23). As such, adding regular sauna bathing to exercise could potentially yield even greater benefits than regular exercise alone. In addition to traditional CVD risk factors, cardiorespiratory fitness (CRF) has also been recently highlighted as a strong predictor of health outcomes (24) and is indicative of functional capacity and overall physical health (25). CRF can be measured directly using maximal testing or estimated via submaximal testing and is a significant prognosticator regardless of the method by which it is derived (26).

Our previously published works showed promising results through sauna use (27), as well as via a combination of exercise followed by sauna (28) in acute responses. Our objective for the current experiment was thus to expand these findings and explore the likelihood of cardiovascular adaptations, using BP and CRF as primary outcomes. We seek to provide fundamental and valuable information to the study of heat therapy and sauna use and its potential as a lifestyle intervention that could be prescribed alongside exercise effectively.

As such, we conducted an 8-wk multi-arm randomized controlled trial (RCT) using the current recommended guidelines on physical activity, in a population with CVD risk factors. The primary focus was to compare the cardiovascular adaptations of regular exercise alone (EXE) to regular exercise and sauna bathing (EXS), with a sedentary control (CON) group serving as a comparator against the EXE group to ascertain the efficacy of the 8-wk exercise intervention. To the best of our knowledge, this is the first multi-arm RCT investigating the long-term effects of exercise and sauna use in a nonathletic and nonclinical general population.

## MATERIALS AND METHODS

**Figure 1.**
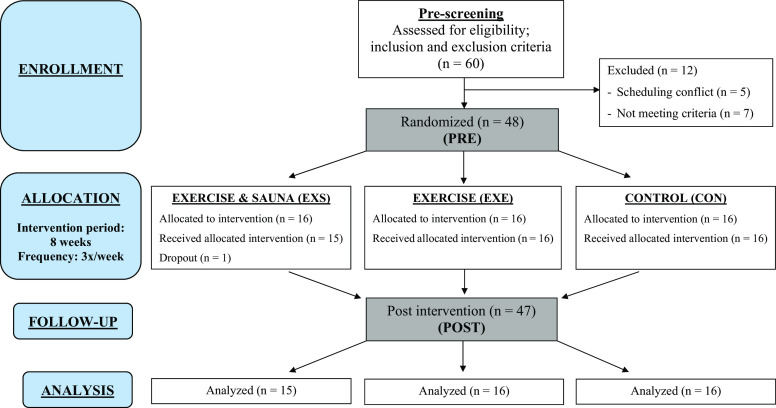
Experimental design (adapted and modified according to CONSORT guidelines template). *n*, number of human volunteers/participants.

A multi-arm, parallel-group (allocation ratio 1:1:1) RCT (Unique identifier: NCT04540718) was conducted in accordance with Consolidated Standards of Reporting Trials (29) guidelines (Fig. 1). The institutional review board of the Central Finland Hospital District ethical committee, Jyväskylä, Finland, approved this study (Dnro 3 U/2019). All participants provided written informed consent. The data that support the findings of this study are available to researchers upon reasonable request to the corresponding author.

### Study Population

Female and male participants between 30 and 64 yr were recruited through medium-to-large organizations (City council of Jyväskylä, Jyväskylä Energy, Central Finland hospital) via email. The inclusion criteria of study participants consisted of a sedentary lifestyle and at least one traditional CVD risk factor. Sedentary lifestyle was identified as having a desk-bound job and less than 30 min of total physical activity per week. The CVD risk factors were elevated cholesterol, family history of coronary heart disease (CHD), hypertension, obesity, and smoking.

Total cholesterol level >239 mg/dL was considered elevated. Family history of CHD was positive if father (<55 yr) or mother (<65 yr) had premature CHD. Prestudy resting systolic BP (SBP) >139 mmHg and/or diastolic (DBP) >89 mmHg was considered elevated (30). Obesity was defined as body mass index (BMI) >30 kg/m^2^. Exclusion criteria were *1*) sauna bathing more than once a week within the past 6 mo, *2*) commuting to work via activities such as running or cycling, *3*) previous CHD and/or diabetes, and *4*) any diagnosed and/or symptomatic CVD, musculoskeletal injury, or any other physical or mental condition within 6 mo before the commencement of the study. Participants were also excluded if they had resting SBP <100 mmHg or >159 mmHg, BMI over 40 kg/m^2^, or if they were on any CVD medication.

Before the trial commencement, participants (*n* = 60) attended an information session where they were briefed about the research purposes, measurement procedures, and intervention period. Five participants dropped out. Subsequently, a prescreening session was conducted to collect baseline information (anthropometric data, resting electrocardiogram, and brachial BP) and ensure that the remaining 55 met the study eligibility based on the inclusion and exclusion criteria. Seven participants who did not meet the criteria were excluded, leading to a final sample size of 48 participants.

### Randomization and Design

After the successful completion of prescreening procedures, participants were randomized into the EXS, EXE, or the CON group (Fig. 1). The randomization sequence was created using Excel 2016 (Microsoft, Redmond, WA) with a 1:1:1 allocation using simple randomization with stratification by a researcher with no clinical involvement in the trial. Biological sex was used for stratification, as there was a disproportionate number of female to male participants. Forty-eight participants (42 females, 6 males) were enrolled into the trial by the corresponding author. Participants were assigned to their respective interventions (16 per group, 14 females, and 2 males) by a member of the research team who was uninvolved with the data collection and analysis process. To ensure that the statistical analyses were nonbiased, the statistician was blinded to the assignment and completely uninvolved in the participant recruitment and data collection processes.

Participants in the CON group were informed that a similar 8-wk supervised exercise training program would be offered to them after the trial. This was done to minimize dropout rates and increase adherence to preexisting lifestyle and physical activity habits during the trial period to reduce potential confounding factors. The study consisted of two measurement days completed by all groups and an 8-wk intervention for the EXS and EXE groups. Participants recorded and submitted a food diary the day before their preintervention (PRE) measurement. This was returned to them 48 h before the postintervention (POST) measurement, and they were carefully instructed to follow the same food intake before their measurement days. All participants were reminded regularly to maintain their regular daily activities and diet to minimize the possible influence of external variables on the outcome measures.

**Figure 2.**
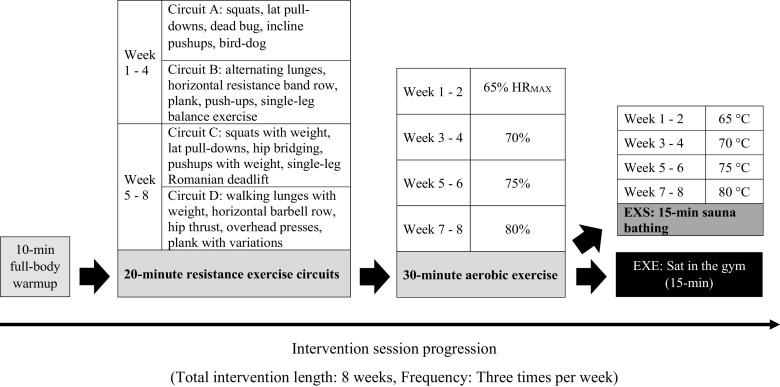
Details of the intervention. Loads were increased for resistance exercises when participants were able to complete the movement comfortably with good form. More challenging variations for the bodyweight exercises were introduced when the participant completed the basic movement with no noticeable difficulties. For example, resistance bands were used for dead bugs, bird-dog were executed with eyes closed, etc.

The intervention groups exercised three times a week (Monday, Wednesday, and Friday) in the evenings, between 1600 and 2100. Training sessions were carried out in groups of 1–5 participants with two qualified instructors. A predetermined adherence rate of 95% for 24 training sessions was successfully achieved. One participant from the EXS group dropped out during the first week due to undisclosed personal reasons. The exercise intervention was based on the Finnish national exercise guidelines (31), which are adapted from the guidelines of the American College of Sports Medicine (ACSM) and reflect current recommendations (3). Each exercise session lasted 60 min and was performed in the following order: a 10-min full-body warm-up, 20 min of resistance exercise, and 30 min of aerobic exercise. Details of the intervention are shown in Fig. 2.

Resistance training was a mixture of body weight and basic resistance training exercises. Starting loads for each resistance exercise were determined individually on a separate day before the intervention. The exercises were performed in a circuit fashion, with the aim of providing a full-body workout. Each circuit consisted of five movements, and each movement was performed for 45 s with a 15-s break between them. Completion of a circuit took 5 min, followed by a 1-min break. The circuits were completed three times each session. *Circuits A* and *B* were used alternately in each training for the first 4 wk, and *circuits C* and *D* were used in the final 4 wk. Harder variations of body weight movements and greater resistance exercise loads for each individual were introduced as performance improved, based on the assessment of the exercise instructors.

Aerobic exercise was performed using Monark cycle ergometers (Monark 828 E, Varberg, Sweden). Individual maximum heart rates were calculated (32) and used thereafter to prescribe aerobic exercise intensity, starting from 65% of maximum heart rate with a fortnightly increase of 5%. Aerobic exercise heart rate was closely monitored and verified every 5 min. Participants maintained a constant pedaling frequency of 65–70 revolutions per minute (rpm), while the magnetic resistance of the bike ergometer was adjusted to achieve the required exercise intensity. After aerobic exercise, participants in the EXS group proceeded to the sauna room, whereas those in the EXE group waited in the gym until the participants in the EXS group completed 15 min of sauna exposure. The temperature of sauna exposure started from 65°C and was increased by 5°C fortnightly and was monitored and recorded every minute via a commercially available wireless thermometer unit (Wireless thermometer 7410; Suomen Lämpömittari Oy, Helsinki, Finland). Relative humidity of the sauna room was between 10% and 20%. Participants were allowed to leave the sauna at any time if they felt uncomfortable, but all participants in the EXS group completed all 15 min of every postexercise sauna exposure successfully without leaving the sauna room.

### Measurement of Outcomes

All measurements (PRE and POST) took place in the exercise and health laboratory, at the Faculty of Sport and Health Sciences, of the University of Jyväskylä. The primary outcomes were estimated relative maximal oxygen uptake (V̇o_2max_) as a measure of CRF and brachial BP. The CRF tests were completed in the evening between 1600 and 2000, and participants were instructed to refrain from heavy physical activity for 48 h and abstain from alcohol and nicotine for 12 h before all CRF tests. A multistage test similar to the YMCA submaximal bicycle test (33) was used. The test consisted of four stages of incremental submaximal workloads lasting 4 min each. Cadence was kept at 50–60 rpm (34), and heart rate was measured via a heart rate monitor (Polar V800; Polar Electro Oy, Kempele, Finland). After the test was completed, a regression line was plotted using the four points corresponding to each stage and extrapolated to the maximal heart rate. A perpendicular line was subsequently formed down to the *x*-axis and absolute oxygen uptake was read off from the graph (33, 34). From the resulting value, relative V̇o_2max_ was then calculated using individual body mass.

Two separate brachial BP measurements were taken on the right upper arm using automated oscillometric devices. The first measurement was taken with the Omron HEM-7320-LA (Omron Healthcare Co., Ltd, Kyoto, Japan), followed by the Arteriograph (TensioMed, Budapest, Hungary) after a 10-min rest in a supine position, according to the established guidelines (30, 35). If the difference in SBP between the two measurements was larger than 10 mmHg, another measurement was taken after a 5-min rest. The two measured values that differed the least were averaged and used for applanation tonometry analysis. Arterial stiffness indices of pulse-wave velocity (PWV) and augmentation index (AIx) as secondary outcomes were recorded noninvasively with the Pulsepen device (DiaTecne s.r.l., Milan, Italy; www.pulsepen.com) according to published guidelines (36). BP and tonometer measurements were taken by a single trained operator to ensure consistency. Intraclass correlation coefficient (ICC) estimates and their 95% CI were calculated using statistical software R (36), based on a mean-rating (*k* = 2), absolute-agreement, two-way mixed-effects model (ICC 2.1: 0.81 with 95% CI = 0.77–0.85, SE = 0.4).

Body composition measurements and blood samples were collected in the morning between 0630 and 0930 in fasted conditions. Participants were instructed to abstain from food, drinks, alcohol, and nicotine for 12 h and to refrain from heavy physical activity for 48 h before all measurements. Body composition was determined using dual-energy X-ray absorptiometry, and venous blood samples were collected by a qualified technician from the antecubital vein into Vacuette SST 6-mL tubes using sterile needles. The sample was centrifuged for 10 min at 2,000 rpm after which serum was removed and stored at −80°C until chemical analyses. Serum samples were subsequently analyzed with colorimetric assay, Konelab 20 analyzer (Thermo, Vantaa, Finland) to determine total cholesterol levels. Sensitivities and coefficients of variation (with-in-assay CV % average) were 3.86 mg/dL and 1.00%.

### Statistical Analyses

Categorical variables are presented as number (%), whereas continuous variables are presented as means ± SD; 95% CIs are presented where appropriate. Statistical power of 80% with α = 0.05 for a difference of one standard deviation in means can be achieved with *n* = 15.68 per group when a two-tailed independent samples *t* test is used. Thus, the final sample size of 15, 16, and 16 (EXS, EXE and CON, respectively, with two males per group) in the three groups was expected to be adequate to detect most clinically significant differences.

Distributions of responses were tested for normality with the Shapiro–Wilk test for each group separately. After the family-wise error rate was controlled by using a Bonferroni correction, the null hypothesis of normality was not rejected for any variable of any group. Between-group differences PRE and POST intervention were analyzed using independent *t* tests. The comparisons were done between CON and EXE groups and between EXE and EXS groups. The level for statistical significance was set at *P* ≤ 0.05. For completeness, we also fitted a mixed linear model for all three groups and each response variable. The details and results of these exploratory analyses are shown in Supplemental Table S1 (see https://doi.org/10.6084/m9.figshare.20160479.v1). The calculations were implemented with the statistical software R (37), with graphics done using the ggplot2 package (38).

## RESULTS

### Characteristics of Participants

The characteristics of the participants are presented in Table 1. The three most commonly present traditional CVD risk factors were obesity (54%), family history of CHD (37%), and elevated BP (35%). Baseline V̇o_2max_, SBP, and DBP were 28.3 ± 5.6 mL/kg/min, 133 ± 12 mmHg, and 79 ± 10 mmHg, respectively. No sex-based or race/ethnicity-based differences were present.

**Table 1.** Baseline participant characteristics

Characteristics	Total *n* = 47	Control *n* = 16, 14 Females	Exercise Only *n* = 16, 14 Females	Exercise + Sauna *n* = 15, 13 Females
Age, yr	49 ± 9	49 ± 8	51 ± 9	47 ± 8
Body mass, kg	89.0 ± 14.3	86.5 ± 15.6	87.3 ± 13.0	93.5 ± 13.2
Body mass index, kg/m^2^	31.3 ± 4.1	31.1 ± 4.7	31.3 ± 4.2	32.2 ± 3.6
Estimated V̇o_2max_, mL/kg/min	28.3 ± 5.6	30.1 ± 4.8	29.4 ± 5.7	26.4 ± 6.3
Systolic BP, mmHg	133 ± 12	129 ± 9	134 ± 14	134 ± 14
Diastolic BP, mmHg	79 ± 10	78 ± 5	79 ± 11	80 ± 13

Risk Factors*	Number (%)	Number/Group Total		
Obesity (BMI >30 kg/m^2^)	25 (54%)	8/16	9/16	8/15
Family history of CHD	18 (38%)	8/16	5/16	5/15
Elevated BP	16 (35%)	7/16	5/16	4/15
Elevated cholesterol (>239 mg/dL)	10 (22%)	4/16	3/16	3/15
Smoker (history of smoking)	6 (13%)	2/16	2/16	2/15

Values are means ± SD. No significant differences were found at baseline between groups for all the parameters. BMI, body mass index; BP, brachial blood pressure; CHD, coronary heart disease; V̇o_2max_, maximal oxygen consumption. *Number of participants with one, two, and three risk factors was 23, 20, and 4, respectively. No participant had more than three risk factors.

### Exercise versus Control

Significant PRE-POST differences were found between the control (CON) and exercise (EXE) groups for V̇o_2max_ and fat mass. Comparatively, EXE had greater increases in V̇o_2max_ and decreases in fat mass. No significant differences were found for BP, arterial stiffness indices, and total cholesterol (Table 2). Female-only data are found in Supplemental Table S2 (see https://doi.org/10.6084/m9.figshare.19575979).

**Table 2.** PRE-POST comparison of means between the EXE and CON groups

Outcome Variable	EXE (*n* = 16, 14 Females)		CON (*n* = 16, 14 Females)			
	PRE	POST	PRE	POST	Mean Difference, 95% CI	*P* Value
Estimated V̇o_2max_, mL/kg/min	29.4 ± 5.7	32.0 ± 6.6	30.1 ± 4.8	26.8 ± 4.6	6.2 (4.1, 8.3)	0.00000211
SBP, mmHg	134 ± 14	134 ± 14	130 ± 9	130 ± 10	0.5 (−4.6, 5.6)	0.841
DBP, mmHg	79 ± 11	80 ± 9	79 ± 5	82 ± 6	−1.9 (−5.5, 1.7)	0.295
Fat mass, kg	37.8 ± 10.5	36.5 ± 10.1	38.0 ± 12.4	38.0 ± 12.3	−1.3 (−2.3, -0.3)	0.0125
Total cholesterol, mg/dL	203 ± 34	208 ± 30	215 ± 34	211 ± 29	12 (−8, 27)	0.215
PWV, m/s	9.2 ± 1.7	9.2 ± 1.4	8.5 ± 1.5	8.7 ± 2.4	−0.2 (−1.2, 0.8)	0.662
AIx, %	16.1 ± 11.9	17.3 ± 10.0	15.5 ± 11.0	15.4 ± 8.7	1.2 (−6.5, 8.9)	0.760

Values are means ± SD. Data were analyzed using independent *t* tests. AIx, augmentation index; CI, confidence interval; CON, control; DBP, brachial diastolic blood pressure; EXE, exercise; PRE, preintervention; POST, postintervention; PWV, pulse-wave velocity; SBP, brachial systolic blood pressure; V̇o_2max_, maximal oxygen consumption.

### Exercise and Sauna versus Exercise

**Figure 3.**
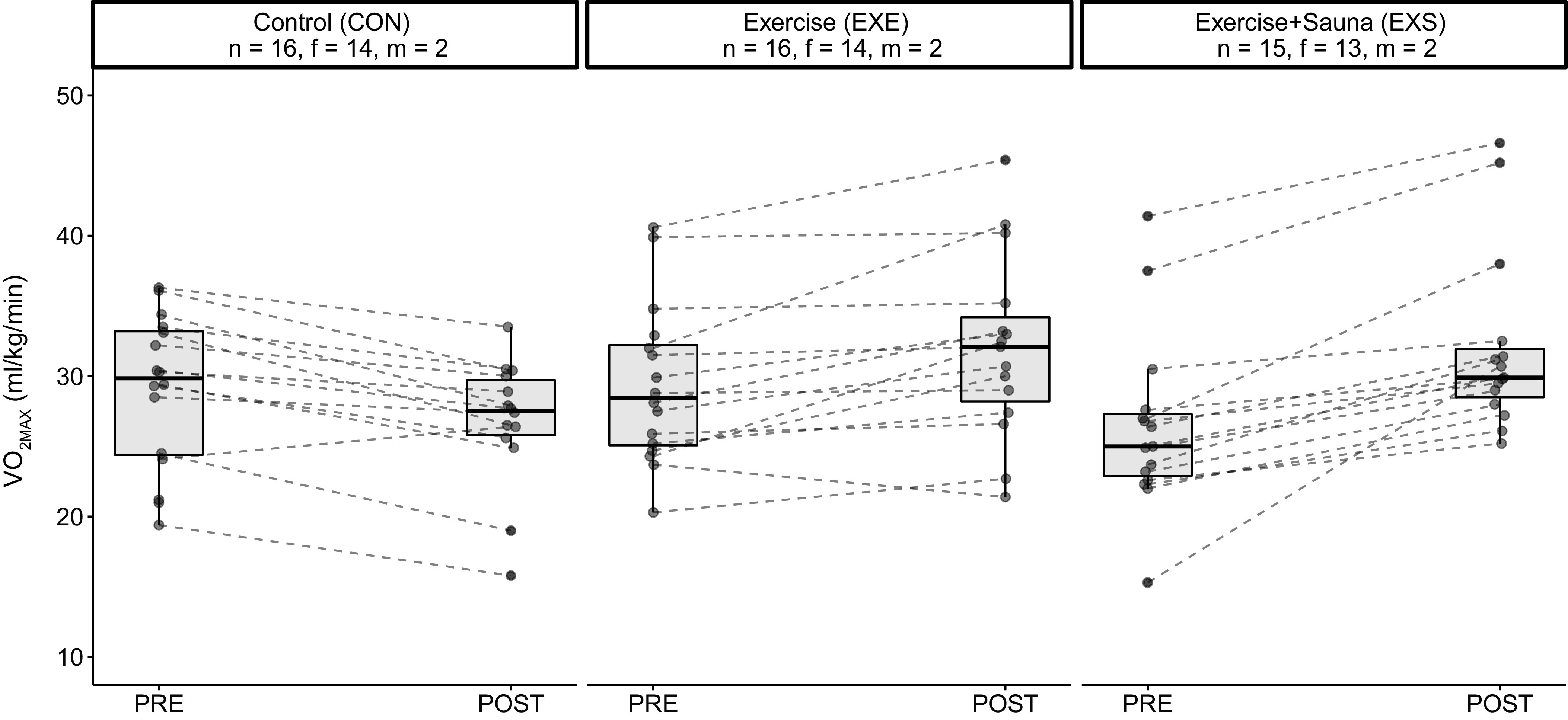
Graphical representation of the PRE-POST changes in CRF (relative V̇o_2max_) of the three groups. CRF, cardiorespiratory fitness; f, female; m, male; PRE, preintervention; POST, postintervention.

**Figure 4.**
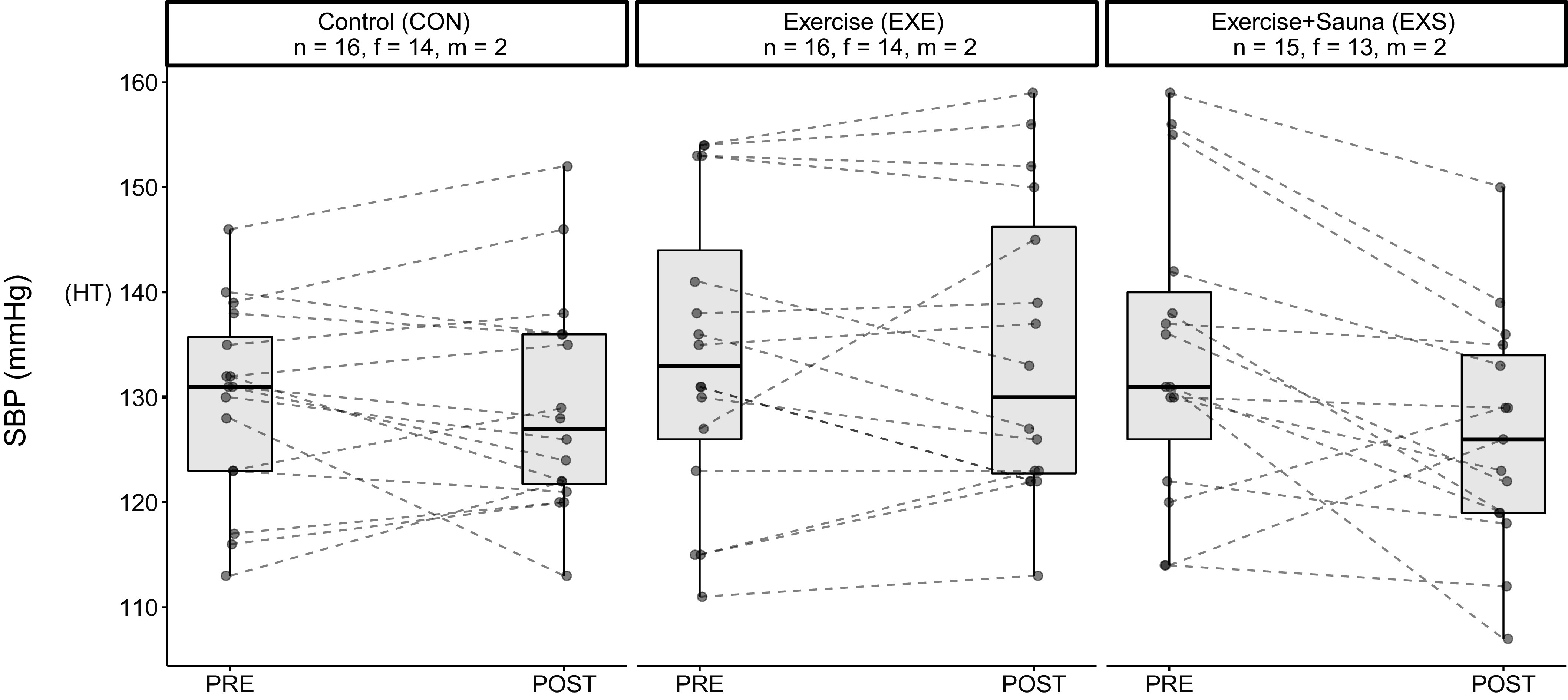
Graphical representation of the PRE-POST changes in SBP of the three groups. HT, Grade 1 hypertension classification; f, female; m, male; PRE, preintervention; POST, postintervention; SBP, systolic blood pressure.

**Figure 5.**
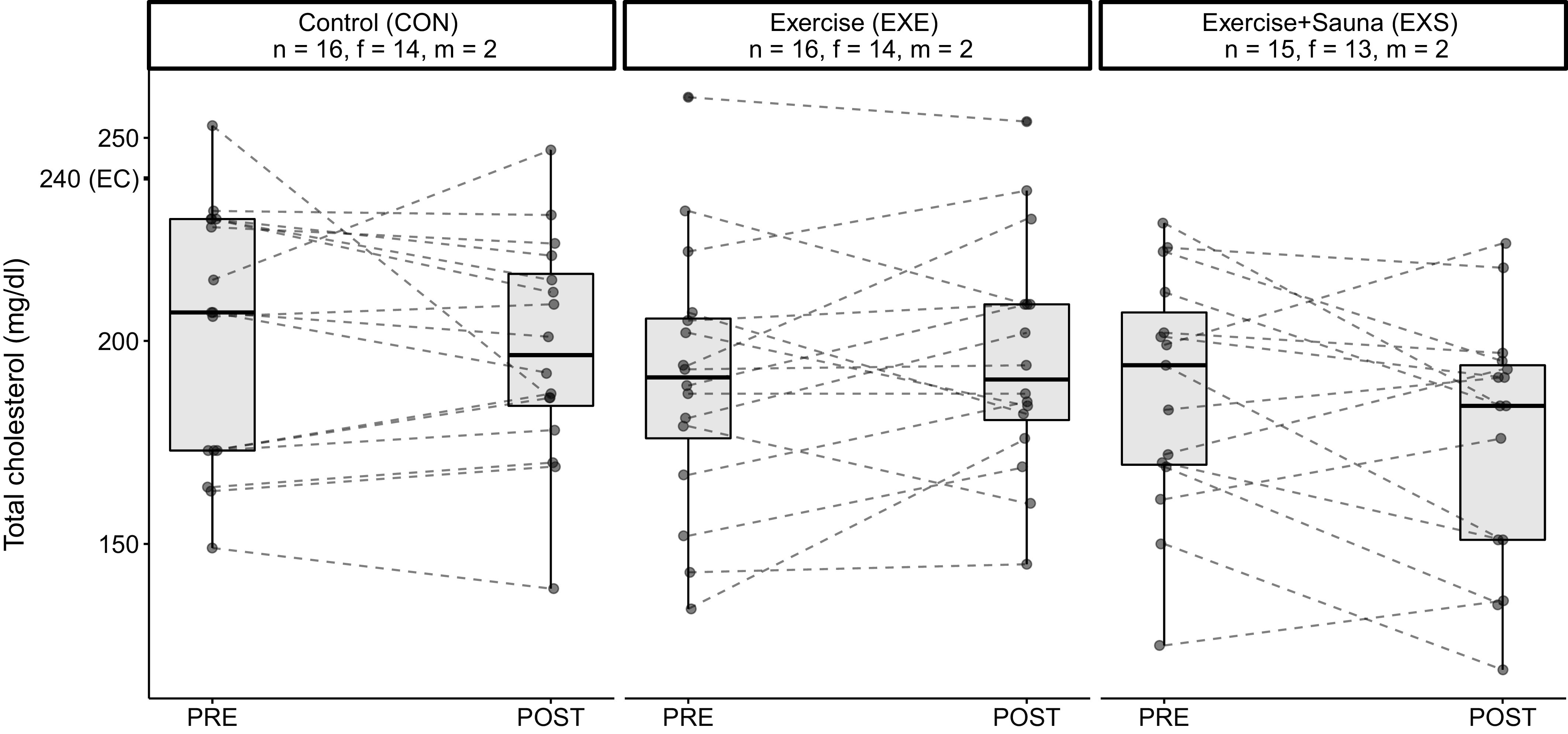
Graphical representation of the PRE-POST changes in total cholesterol levels of the three groups. EC, elevated cholesterol; f, female; m, male; PRE, preintervention; POST, postintervention.

PRE-POST differences in V̇o_2max_, SBP, and total cholesterol levels were significant between the EXE and exercise and sauna (EXS) groups (Table 3). Specifically, V̇o_2max_ was greater (Fig. 3), whereas SBP (Fig. 4) and total cholesterol levels (Fig. 5) were lower in the EXS than the EXE group after the 8-wk intervention period. There were no differences in any other outcome variables. Female-only data are found in Supplemental Table S3 (see https://doi.org/10.6084/m9.figshare.19576030).

**Table 3.** PRE-POST comparison of means between the EXS and EXE groups

Outcome Variable	EXS (*n* = 15, 13 Females)		EXE (*n* = 16, 14 Females)			
	PRE	POST	PRE	POST	Mean Difference, 95% CI	*P* Value
Estimated V̇o_2max_, mL/kg/min	26.4 ± 6.3	32.0 ± 6.4	29.4 ± 5.7	32.0 ± 6.6	2.7 (0.2, 5.3)	0.0343
SBP, mmHg	134 ± 14	126 ± 11	134 ± 14	134 ± 14	−8.0 (−14.6, −1.4)	0.0198
DBP, mmHg	80 ± 13	80 ± 14	79 ± 11	80 ± 9	−0.6 (−6.0, 4.8)	0.823
Fat mass, kg	39.6 ± 8.2	37.7 ± 8.5	37.8 ± 10.5	36.5 ± 10.1	−0.6 (−1.9, 0.7)	0.339
Total cholesterol, mg/dL	200 ± 32	188 ± 33	203 ± 34	208 ± 30	−19 (−35, 0)	0.0467
PWV, m/s	9.6 ± 1.9	9.2 ± 1.7	9.2 ± 1.7	9.2 ± 1.4	−0.4 (−1.1, 0.3)	0.249
AIx, %	17.7 ± 10.6	12.6 ± 14.1	16.1 ± 11.9	17.3 ± 10.0	−6.3 (−14.8, 2.2)	0.142

Values are means ± SD. Data were analyzed using independent *t* tests. AIx, augmentation index; CI, confidence interval; DBP, brachial diastolic blood pressure; EXE, exercise; EXS, exercise + sauna; PRE, preintervention; POST, postintervention; PWV, pulse wave velocity; SBP, brachial systolic blood pressure; V̇o_2max_, maximal oxygen consumption.

## DISCUSSION

In this multi-arm RCT, we compared the effects of an 8-wk exercise and sauna intervention (EXS) to regular exercise without sauna (EXE), using a sedentary population with relatively low physical activity levels and at least one traditional CVD risk factor. A control group (CON) was included to validate the efficacy of the exercise intervention. Our results show improvements in CRF based on the estimated V̇o_2max_ and lower fat mass for the EXE group compared with the CON group. More importantly, the EXS group demonstrated a greater increase in CRF, and greater decreases in SBP and total cholesterol levels, when compared with the EXE group.

To a reasonable extent, the differences seen between the CON and EXE groups were expected. Physical activity guidelines supported by research evidence suggest that 150 min of moderate-intensity exercise per week is sufficient to induce beneficial health adaptations (4). As such, our 8-wk exercise intervention was constructed to adhere as closely as possible to the recommendations (31). Every supervised session included a full-body warm-up, followed by resistance, then aerobic exercise. In addition, exercise intensity was progressively increased throughout the 8 wk, for both resistance and aerobic exercise.

The long-term benefits of exercise training on physical health have been well established (3, 39), and regular aerobic training has been shown to improve both body composition (39) and CRF (40) even at relatively lower intensities, which is consistent with the main findings of the current study. Furthermore, it has been well documented that performing resistance exercise before aerobic exercise leads to higher energy expenditure and fat mass loss (41–43), which was how our training sessions were designed. The results from this experiment are in support of the literature and are indicative that the 8-wk exercise intervention provided an adequate stimulus for physiological adaptations to both CRF and body composition.

Despite these adaptations, however, there were no differences in changes to BP and other secondary variables such as arterial stiffness between the EXE and CON groups. This may have been partially due to the length of the present intervention, as training interventions are typically longer in duration. Although cardiovascular adaptations such as arterial remodeling and capillary growth have been well documented to occur within the first few weeks of exercise training (44), this did not appear to be the case. The structure of the exercise training likely contributed to this lack of response in the other variables, as the divergent nature of resistance and aerobic exercise has been reasonably established (45). Moreover, combined resistance and aerobic training have been documented to be less effective than aerobic training alone in reducing arterial stiffness (46) and BP (47).

One of the objectives of the current study was to elucidate the synergistic effects of sauna exposure and exercise on the primary variables of BP and CRF, in a sedentary population with traditional CVD risk factors. Specifically, our data suggest that the addition of 15-min sauna exposure, regularly after every exercise session, three times a week for 8 wk was able to improve CRF, SBP, and total cholesterol levels significantly, when compared with performing the same exercise intervention alone. Indeed, previous studies have shown that sauna exposure is an effective additive tool to an exercise program for both clinical (13, 48) and athletic populations (10, 49).

Studies have found positive acute cardiovascular responses from the use of passive heat (50), sauna exposure (27, 51), and sauna exposure with exercise (52, 53). A recent study also showed that postexercise sauna exposure had an augmentative effect, thereby increasing the overall training stress (54). However, to our best knowledge, this is the first multi-arm RCT investigating the cardiovascular and health effects of long-term sauna exposure in the general population with CVD risk factors. The current results suggest that the addition of regular sauna exposure was able to increase CRF and led to a decrease in SBP when comparing the EXS and EXE groups.

Heat acclimation studies have shown the efficacy of using heat to improve aerobic fitness (55, 56). Moreover, the use of heat has been shown to induce a greater level of acute physiological strain and cellular response at a lower relative workload than hypoxia (57). Therefore, it is more likely that the present cardiovascular adaptations seen in this study may be the result of functional enhancements rather than structural changes in the arteries (58), as there were no significant changes to PWV and AIx as measures of arterial stiffness. Nevertheless, this needs to be further investigated, as heat shock proteins, muscle endothelial nitric oxide synthase content, and capillary density were not measured in the present study.

It is worth noting that CRF adaptations to passive heat exposure in the form of sauna bathing have yet to be thoroughly investigated mechanistically. However, cardiovascular adaptations to heat acclimation have been well documented (59), which provides us with a vital framework that may explain the difference in CRF between the EXE and EXS groups. Exercise training and passive heat have been shown to have additive effects that lead to improved myocardial contractility, over exercise training or heat alone, in animal models (60). Eight weeks of heat acclimation has been shown to improve myocardial compliance, rendering it more efficient (60, 61). It is thus plausible that cardiovascular stability may have been augmented by the addition of regular sauna exposure to exercise, and that functional, rather than morphological adaptations were responsible for the differences in CRF between the intervention groups, particularly in the absence of changes to arterial stiffness parameters. Moreover, infrared sauna therapy (13) has also been shown to augment increases in CRF via functional changes, which is consistent with our present findings.

A recent systematic review showed that compared with controls, heat therapy decreased both SBP and DBP by an average of 4 mmHg (23). Based on the latest guidelines on BP (30, 35), it was postulated that a reduction in BP by ∼5 mmHg would improve individual BP categorization (23, 62). Although our study did not find differences in DBP between interventions, SBP levels for the EXS group were 8 mmHg lower than in the EXE group postintervention, which is almost an entire BP category. This is a clinically important new finding to highlight, as a recent meta-analysis (63) reported a nearly linear relationship between a 5-mmHg decrease in SBP and a lowered risk of all-cause mortality across all BP categories.

Although higher resting BP before an intervention has been suggested as a potential mitigating factor in the therapeutic effects seen in heat-related studies (23), only 35% of the participants in the current experiment had an elevated resting BP. In addition, there was no difference in the PRE values of SBP and DBP between all three groups. One mechanism that may have contributed to the lower SBP in the EXS group was the concomitant lowering of total cholesterol levels. This agrees with the findings from an earlier study (64), which found that heat therapy was able to improve the blood lipid profile, specifically total cholesterol levels, in a sedentary obese population. In addition, there were improvements to BP but no changes to BMI or body composition, which is remarkably comparable to the findings from our current study. Even though the authors used hot water immersion as opposed to the sauna, there were similarities between our experimental designs worth noting, such as the intervention period and the frequency of exposure. These are crucial factors to consider for future research in the area, as has been pointed out by several others (23, 58).

Some limitations of this study ought to be noted. The trial lacked a sauna-only group, which would have allowed us to determine if the benefits seen could have been solely attributable to the sauna. Diet of the participants was not controlled for during the trial, which could have influenced the results. However, participants followed the same diet a day before each measurement was taken, which improves the consistency and reliability of our data. Maximal oxygen consumption was not measured directly, but the indirect method better suited the study population and added external validity. The study sample had only six male participants. However, we addressed this issue using stratified randomization and included separate tables of results (Supplemental Tables S2 and S3, Supplemental Fig. S1; see https://doi.org/10.6084/m9.figshare.19582801.v1, Supplemental Fig. S2; see https://doi.org/10.6084/m9.figshare.19582813.v1, Supplemental Fig. S3; see https://doi.org/10.6084/m9.figshare.19582822.v1) that excludes the males for a more accurate and complete perspective. Moreover, insufficient female data have been a long-standing problem in scientific research; therefore, this could be viewed as a strength, rather than a limitation of this study.

Indeed, our study does have several strengths. We extended the findings of our earlier research in the acute setting (28, 65), with an 8-wk interventional study using a sedentary population, who were nonfrequent sauna users, to investigate the complementary effects of exercise followed by sauna exposure. Body composition was determined using dual-energy X-ray absorptiometry, while arterial stiffness and BP measurements adhered closely to established guidelines (30, 35, 36). Compliance of the intervention groups was excellent, with only 2 participants each missing a single session out of 24. All other participants completed the 24 sessions successfully with only 1 dropout for the entire study. Moreover, a statistician that was blind to the intervention assignment performed the data analysis using coded variables.

In conclusion, regular exercise using the recommended guidelines three times a week, for 50 min each time, can effectively improve CRF and body composition. The addition of a regular 15-min typical Finnish sauna after exercise supplemented the gains in CRF, reductions in SBP, and lowered total cholesterol levels considerably. Future research should adopt a more systematic approach in the study of heat exposure and seek to understand the optimal exposure durations, frequencies, modalities, and temperatures for various beneficial adaptations.

### Perspectives and Significance

The design of this experiment allowed us to ascertain to a reasonable extent the additive effect of regular sauna exposure to exercise on cardiovascular health outcomes such as BP and CRF. These beneficial changes seen are promising, given that the essential methodological parameters of sauna exposure, such as duration and frequency were not only relatively short and tolerable, but practically feasible and replicable as well. Taken into context with mechanistic studies from molecular physiology, this is indicative of the noteworthy potential that passive heat therapy has. In addition, this study opens up opportunities to investigate shorter bouts of regular exercise in conjunction with sauna use and lends support for regular sauna bathing to be a possible therapeutic alternative, particularly for those with compromised exercise capacities, and possibly other rehabilitation settings as well. Sauna bathing is a safe and simple lifestyle modification and steps should be taken to make it more accessible worldwide.

## DATA AVAILABILITY

The data that support the findings of this study are available to researchers upon reasonable request to the corresponding author.

## SUPPLEMENTAL DATA

10.6084/m9.figshare.20160479.v1 Supplemental Table S1; https://doi.org/10.6084/m9.figshare.20160479.v1.

10.6084/m9.figshare.19575979.v1 Supplemental Table S2 https://doi.org/10.6084/m9.figshare.19575979.v1.

10.6084/m9.figshare.19576030.v1 Supplemental Table S3 https://doi.org/10.6084/m9.figshare.19576030.v1.

10.6084/m9.figshare.19582801.v1 Supplemental Fig. S1 https://doi.org/10.6084/m9.figshare.19582801.v1.

10.6084/m9.figshare.19582813.v1 Supplemental Fig. S2 https://doi.org/10.6084/m9.figshare.19582813.v1.

10.6084/m9.figshare.19582822.v1 Supplemental Fig. S3: https://doi.org/10.6084/m9.figshare.19582822.v1.

## GRANTS

The Finnish Cultural Foundation (SKR) partially funded the corresponding author’s salary (Grant No.: 00190620 to E. Lee).

## DISCLOSURES

No conflicts of interest, financial or otherwise, are declared by the authors.

## AUTHOR CONTRIBUTIONS

E.L. conceived and designed research; E.L. and I.K. performed experiments; J.K. analyzed data; E.L., J.P.A., E.A.H., and J.A.L. interpreted results of experiments; J.K. prepared figures; E.L. and J.K. drafted manuscript; E.L., J.K., J.P.A., E.A.H., P.W., S.K.K., and J.A.L. edited and revised manuscript; E.L., I.K., J.K., J.P.A., E.A.H., P.W., S.K.K., and J.A.L. approved final version of manuscript.
